# Vortex Chain in a Resonantly Pumped Polariton Superfluid

**DOI:** 10.1038/srep09230

**Published:** 2015-03-18

**Authors:** T. Boulier, H. Terças, D. D. Solnyshkov, Q. Glorieux, E. Giacobino, G. Malpuech, A. Bramati

**Affiliations:** 1 Laboratoire Kastler Brossel, UPMC-Sorbonne Universités, CNRS, ENS-PSL Research University, Collège de France, 4, place Jussieu Case 74, F-75005 Paris, France; 2 Institut Pascal, PHOTON-N2, Université Clermont Auvergne, Blaise Pascal University, CNRS, 24 Avenue des Landais, 63177 Aubière Cedex, France

## Abstract

Exciton-polaritons are light-matter mixed states interacting via their exciton fraction. They can be excited, manipulated, and detected using all the versatile techniques of modern optics. An exciton-polariton gas is therefore a unique platform to study out-of-equilibrium interacting quantum fluids. In this work, we report the formation of a ring-shaped array of same sign vortices after injection of angular momentum in a polariton superfluid. The angular momentum is injected by a *ℓ* = 8 Laguerre-Gauss beam. In the linear regime, a spiral interference pattern containing phase defects is visible. In the nonlinear (superfluid) regime, the interference disappears and eight vortices appear, minimizing the energy while conserving the quantized angular momentum. The radial position of the vortices evolves in the region between the two pumps as a function of the density. Hydrodynamic instabilities resulting in the spontaneous nucleation of vortex-antivortex pairs when the system size is sufficiently large confirm that the vortices are not constrained by interference when nonlinearities dominate the system.

Quantized vortices have been extensively investigated in different fields of physics, such as superconductivity^1^, matter-wave superfluids^2^ and nonlinear optics^3^. In this context, half-light, half-matter polariton fluids offer an unprecedented opportunity to study the quantum-fluid aspects of interacting photons, a rich field at the interface of condensed matter and quantum optics. The recent discovery of polariton Bose-Einstein condensation in semiconductor microcavities^4^,^5^,^6^ has renewed the possibilities of observing superfluidity in quantum fluids of light^7^. As a consequence, the understanding of the mechanism of vortex nucleation and vortex dynamics in such systems becomes particularly important. Vortices are natural topological solutions in interacting photonic systems^8^,^9^. Indeed, the nucleation of vortices^10^,^11^,^12^,^13^,^14^ and half-vortices^15^,^16^ in polariton fluids have been reported in several experiments.

Several schemes to produce vortices and vortex lattices have been proposed and implemented. Liew *et al*^17^ predicted the formation of regular triangular lattices and of Penrose triangular lattices with coherently pumped polariton condensates, while Gorbarch and co-workers^18^ proposed to create robust half-vortex lattices in the optical parametric oscillator (OPO) scheme. Spontaneous self-ordered vortex-antivortex pairs have been recently reported in Refs. 19, 20 and Hivet *et al*^21^ experimentally demonstrated the formation of vortex-antivortex lattices in square and triangle trapping potentials. Nevertheless, none of these schemes allow the effective transfer of a global angular momentum, therefore preventing the spontaneous nucleation of same-sign vortex lattices.

In this work, as a first step towards the physics of rotating polariton quantum fluids, we theoretically propose and experimentally implement a scheme that generates stable chains of same-sign vortices in a coherently driven polariton superfluid. The global angular momentum is injected by mean of a Laguerre-Gauss (LG) laser beam with orbital angular momentum *ℓ*. Note that self-ordered, same-sign vortex lattices have been theoretically predicted in Ref. 22. However, in this ideal, disorder-free scheme, an important experimental limitation arises, as the lattice rotates at very high angular velocities, therefore making time-resolved interferometry measurements a very difficult task. To circumvent the issue of fast rotation in our scheme, an additional Gaussian beam (G) with zero angular momentum is added at the center of the LG beam. The central Gaussian beam allows to lock the azimuthal position of the vortices. We use a Spatial Light Modulator (SLM) to produce the spatial profile of the resonant pumps. This hologram-based optical technique is very versatile and can be used to generate a wide variety of pumping schemes for polaritons. Based on a variational analysis of the generalized Gross-Pitaevskii equation, we then show that the vortex chain is a consequence of the quantization of the total angular momentum transferred to the superfluid, thus different from a trivial interference pattern imposed by the pumps. The quantum nature of the fluid is also confirmed by the appearance of hydrodynamical instabilities, resulting in the nucleation of vortex–antivortex pairs.

These characteristics are close to those of cold atom condensates^29^,^30^,^31^,^33^, where the angular momentum can be externally transferred either by stirring^32^ or via rotation of the trapping potential. In this case, the vortices nucleate and self-organize into an Abrikosov lattice. In our case, the ring geometry is imposed by the pumps, resulting in an additional constraint. In the context of atomic BECs, the method consisting in transferring angular momentum from photons to atoms was studied as well. In particular Dutton and Ruostekoski^28^ theoretically studied the formation of vortex lattices upon illumination with LG beams, and the injection of angular momentum into atomic BECs with LG beams was achieved^26^,^27^. Interestingly, in the case of polariton fluids the injection method is not strictly identical since the rotating polaritons are directly created by the LG pump, as opposed to injections of angular momentum into a preexistent, non-rotating superfluid.

## Results

### Experimental setup

**Figure 1:**
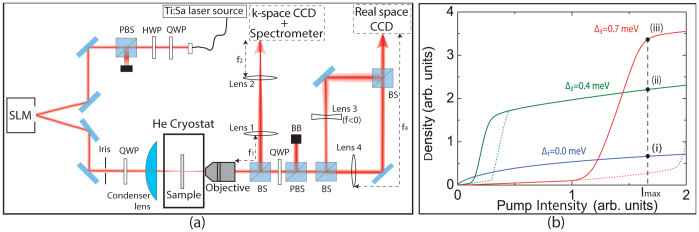
Setup and bistability. (a) - Experimental setup scheme. The pumps are prepared with a pure-phase SLM and sent on the sample. The sample emission is collected and treated for detection in real space, momentum space and energy. (b) - Scheme of the different regimes of bistability used (only the curve obtained when decreasing the density, shown in thick line, is used). A stronger bistability (higher Δ) provides higher polariton densities for high pump intensities. *I*_max_ indicates the pump peak intensity.

We consider a planar semiconductor microcavity in the strong coupling regime, where exciton-photon coupled states (i.e. polaritons) are created by a laser excitation. We make use of a CW single mode, frequency-locked Ti:Sa laser to resonantly excite the cavity. Resonant pumping creates a low-density exciton gas, which allows us to neglect interactions between polaritons and the exciton reservoir, in contrast with experiments performed under non-resonant pumping^20^. The laser is tuned to be quasi-resonant with the ground state of the lower polariton (LP) branch (~837 nm), so that the detuning with respect to the bottom of the LP branch is given by Δ = *ω*_laser_ − *ω*_pol_, where *ω*_laser_ and *ω*_pol_ are respectively the pump and bare polariton energies at normal incidence *k* = 0 μm^−1^. A single-mode, polarization-preserving fiber selects the TEM_00_ mode. The polarization is linear (vertical) after the fiber and the remaining polarization fluctuations are cut by a polarizing beam splitter (PBS). As visible in Fig. 1 (a), the collimated laser beam is then sent to a SLM, which allows us to arbitrarily modify the spatial phase profile of the beam.

By programming a specifically designed phase hologram on the SLM, we can create beams with well-defined intensity and phase profiles. We create a coherent superposition of a ring-shaped Laguerre-Gauss (LG) beam of orbital momentum *ℓ* = 8 and a Gaussian (G) beam of zero orbital momentum at the center. Both the spot size and the intensity of the laser beams are fully determined by the hologram. The LG-beam diameter is chosen to be larger than the waist of the G-beam. As a result, their spatial overlap is small, allowing for the interference to be very weak. To avoid spin-dependent phenomena, the (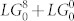) polarization is set to circular with a quarter-wave plate, before being focused on the sample by an aspherical condenser. In Ref. 23, a SLM was used to create a ring shaped condensate, but using non-resonant optical excitation. As a result, the condensate created was in a superposition of two states with opposite angular momentum without the formation of a vortex lattice.

### Sample

The sample is a 2*λ*-GaAs planar microcavity containing three GaAs-InGaAs quantum wells, with a polariton Rabi splitting of 5.1 meV. The cavity finesse is *F* = 3000, which results in a polariton linewidth of about 0.1 meV. The cavity is wedged in one direction, providing a large choice of cavity-exciton detunings by pumping at different positions in the sample. In order to enhance the polariton-polariton interactions, we use a cavity-exciton detuning of *δ* = *ω_C_* − *ω_X_* = 1 meV (*ω_X_*_(*C*)_ is the excitonic (cavity) energy at normal incidence, *k* = 0 μm^−1^), thus increasing the exciton fraction of polaritons and, consequently, the nonlinear effects. The microcavity is cooled down to 5 K in a cryostat and the measurements are taken in transmission. Above a critical value of Δ, a bistable behavior appears^24^. By increasing Δ and working within the upper bistability branch, we increase the polariton density. This is necessary to reach the regime where nonlinearities dominate. We therefore use the polariton bistability to control the density as follows: in the upper bistability branch, at constant pump power *I_p_* = 300 mW, we modify Δ. We consider three different cases: low density (Δ_1_ ≈ 0 meV, point (i) in Fig. 1 (b)), high density far from the bistability threshold (Δ_2_ = 0.4 meV, point (ii) in Fig. 1 (b)) and near the bistability threshold (Δ_3_ = 0.7 meV, point (iii) in Fig. 1 (b)).

### Detection

The detection is simultaneously made in energy and in real- and momentum space. An objective collects the sample emission. CCD cameras are used for direct imaging of the real- and the momentum space, while the energy (wavelength) is measured with a spectrometer. We only collect circularly-polarized light, therefore filtering out any residual spin-flip effect. The polariton phase is measured with an off-axis interferometry setup: a beam splitter divides the real-space image into two parts, one of which is expanded to generate a phase reference beam. The selection of the Gaussian part at the center of the image ensures a flat phase reference, which is used to make an off-axis interference pattern. With this method, the vortex position in the image is independent of the phase of the reference beam^25^. The actual phase map is then numerically reconstructed with a standard off-axis phase detection method.

### Theory

In order to describe the configuration under study, we numerically solve the driven-dissipative Gross-Pitaevskii equation, which in the parabolic approximation reads
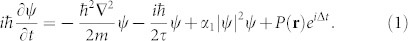
Here, *P*(**r**) = *P_LG_*(**r**) + *P_G_*(**r**) with 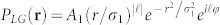 and 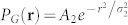; *A*_1_ and *A*_2_ are the amplitudes of the pumping lasers, with *σ*_1_ = 5.0 μm and *σ*_2_ = 3.0 μm. Direct comparison with the experiments is then performed by extracting the steady-state density |*ψ*|^2^ and phase arg(*ψ*).

### Results

**Figure 2:**
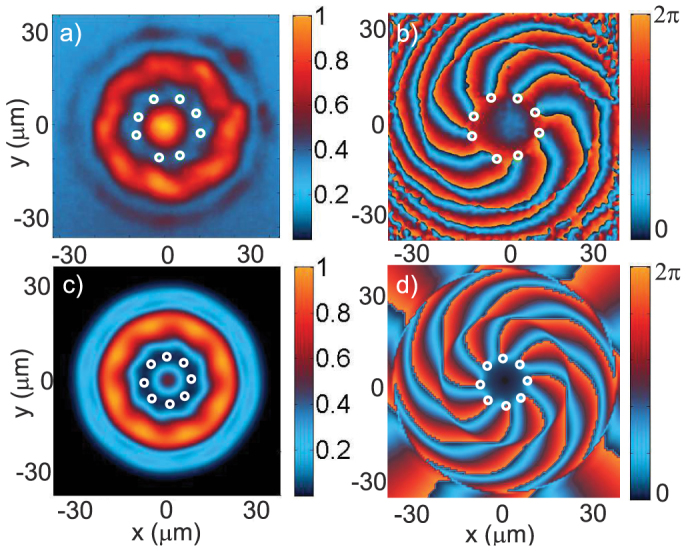
Linear regime. Experimental (above) and theoretical (below) images of the polariton field in the linear regime (low densities, Δ ≈ 0 meV). The intensity |*ψ*|^2^ (normalized to the peak value) is shown on the left panels while the phase arg(*ψ*) is shown on the right panels. Vortices are indicated by white circles both in the intensity and the phase. At low densities the nonlinear interactions are negligible: the pattern is the result of optical interference, fixing the vortex positions.

**Figure 3:**
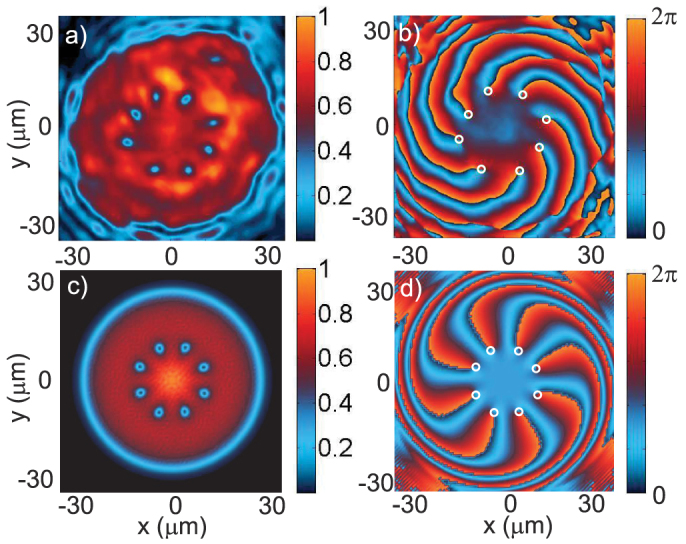
Nucleation of vortices. Experimental (above) and theoretical (below) images of the polariton field in the nonlinear regime (high densities, Δ = 0.4 meV). The intensity |*ψ*|^2^ (normalized by the peak value) is shown on the left panels while the phase arg(*ψ*) is shown on the right panels. Vortices are clearly visible as eight zero-density dips in (a) and (c) and are indicated by white circles in the phase (diagrams (b) and (d)). The polariton density is high enough to enter the superfluid regime and to suppress the pump interference through nonlinear interactions.

In the linear regime (Δ = Δ_1_ ≈ 0 meV), we observe - both experimentally (Fig. 2 a),b)) and theoretically (Fig. 2 c),d)) - a pattern resulting from the optical interference between the LG and G beams. This interference pattern consists in an eight-lobbed spiral (Fig. 2 a),c)) with eight phase singularities (Fig. 2 b),d)). The annular phase singularity chain visible in Fig. 2 is thus imposed by the pump phase. As the density increases, the nonlinear behavior of polaritons is unveiled, with a deformation of the interferences. For Δ = Δ_2_ = 0.4 meV (point (ii) in Fig. 1 (b)), the calculated pattern shown in Fig. 3 (c) is reduced to round-shaped dips of quasi-zero density containing the phase singularities: the eight elementary vortices carrying the injected angular momentum, with a homogeneous density around the dips. In the experiment we observe a ring of same-sign vortices with a density distribution much more uniform than in the linear case, with some residual variations. The vanishing of the interference pattern means that the polariton phase is no more imposed by the pump and is modified through the nonlinear interactions, generating specific features compared to an optical interference pattern. Our observations are consistent with other resonant and non-resonant pumping experiments done in absence of angular momentum^20^,^21^. The formation of a ring of *ℓ* single-charged vortices from a pump with angular momentum *ℓ* is a manifestation of the quantum nature of the polariton fluid, in analogy to the formation of the Abrikosov lattice from a single highly-charged vortex.

**Figure 4:**
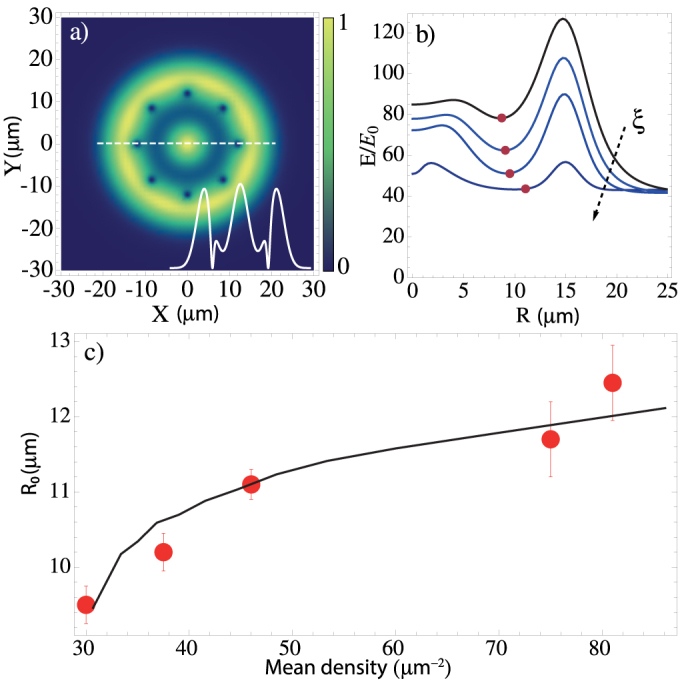
Vortex positions. (a) - Variational wave function in Eq. (2) used to determine the vortex chain radius *R*_0_. The inset shows the radial profile for a cut at *Y* = 0. (b) - Variational energy *E* (in units of 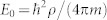) showing a minimum (red dots) for different values of *ξ*. From top to bottom: *ξ* = (3.0, 2.0, 1.0, 0.5) *μ*m. (c) - Average chain radius *R*_0_ as a function of the polariton density, obtained from the variational method (solid line) and the corresponding experimental data (dots).

It is important to remark that the spatial freedom of the vortices provides a quantitative test of the model given below. As predicted by our model, we observe that the vortices in the nonlinear case exhibit a radial phase freedom, while their azimuthal position is locked by the pump phase. Their radial position is modified as the density *ρ* = |*ψ*|^2^ increases. In order to quantify the dependence of the ring radius R_0_ on the relevant experimental parameters, we employ a variational method. We describe the vortex chain solution with the variational ansatz
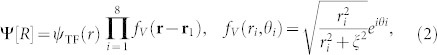
where *ψ*_TF_(*r*) = *ρ*^1/2^[*P*_1_(*r*)/*A*_1_ + *P*_2_(*r*)/*A*_2_] is the Thomas-Fermi density profile induced by the pump, **r***_i_* = *R*(cos*θ_i_*, sin*θ_i_*) is the position of each vortex in the chain, and 
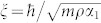
 is the average healing length. In Fig. 4 a), we plot the variational profile given by Eq. (2). The value of *R* that minimizes the energy, *R*_0_, can then be extracted by minimizing the total energy *E*[*R*] = *E*_kin_ + *E*_int_ and taking the physically relevant solution of the condition *δE*/*δR* = 0, where
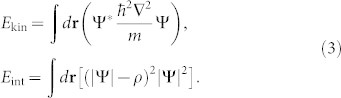
In Fig. 4 b), we plot the energy *E* as a function of *R* for different values of *ξ* ~ *ρ*^−1/2^. We observe that *R*_0_, the value of *R* that minimizes the energy, increases as the value of *ξ* (*ρ*) is decreased. Since *ρ* increases with Δ, *ξ* decreases with Δ; it follows that *R*_0_ increases with Δ. Fig. 4 c) shows *R*_0_ as a function of *ρ* calculated by this method and the comparison with experimental data. As expected, the higher the density, the further from the center the vortices migrate. This behavior and the agreement between the variational method and experiment are a clear indication of the phase freedom obtained when interactions dominate, a feature that is independent from the optical interference.

**Figure 5:**
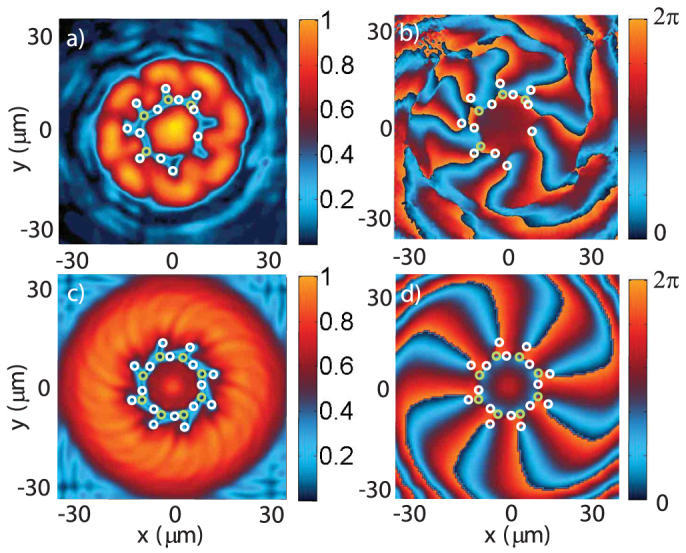
Phase instability. Experimental (above) and theoretical (below) images of the polariton field when the bistability threshold is close to the pump maxima (very high densities, Δ = 0.7 meV). The intensity |*ψ*|^2^ (normalized by the peak value) is shown on the left panels while the phase arg(*ψ*) is shown on the right panels. Vortices are indicated by white circles and antivortices by green circles both in the intensity and the phase. Here only the maxima of the pump are on the upper bistability branch (resonant pumping) and produce a significant polariton density. The nonresonant zone between the LG and G pumps is wide enough for the fluid to hydrodynamically nucleate into vortex-antivortex pairs, while preserving the annular vortex chain.

In the upper bistability branch, the polariton energy is renormalized through self-interaction to the pump energy, so that the pumping is resonant and yields high polariton densities. This is not the case in the lower bistability branch, where the pumping is not resonant and thus inefficient. For large Δ, the low pump intensity regions in the lower bistability branch are off-resonant, as can be seen in Fig. 1 (b). In these regions, non-resonant pumping yields a negligible polariton population and can be considered as a pump-free region. For example, near the threshold, for Δ = Δ_3_ = 0.7 meV (point (iii) in Fig. 1 (b)), a large area between the LG and G pumps is not pumped. Although no polariton is directly injected in this area, the density is not zero due to polaritons propagating from the pumped to the non-pumped area. In this region, the phase is free to evolve, which explains the radial chain expansion. Moreover, when the density in this pump-free area is large enough and when the size of this region is at least of the order of the vortex core ~ *ξ*, we observe the spontaneous nucleation of vortex-antivortex pairs. Four pairs are experimentally visible in Fig. 5 and eight in the theoretical figure. This discrepancy between theory and experiment for the number of pairs is due to the cavity wedge making the experimental cavity anisotropic, a property that is absent in the simulations. The vortex-antivortex pairs form a low-density ring inside the vortex chain described above. This is due to a hydrodynamic instability of the same nature as the one observed in the *ℓ* = 0 case^14^, but here each pair formation is stimulated by the presence of a vortex which acts as a defect. The disorder-free model confirms that the vortex-antivortex pairs are generated by the vortices of the main chain and not by the disorder. This feature also proves that the vortex distribution is not due to optical interference in the superfluid regime, but that it rather evolves with the density.

## Discussion

In the present work, we resonantly inject polaritons with a given total angular momentum and observe the formation of a ring of quantized single-charged vortices. For the first time, a regular ring pattern of elementary vortices of the same sign is reported in a polariton superfluid. In the superfluid regime, the radial position of each vortex is not determined by the pump but rather depends on the polariton density. Experimental and theoretical indications of this behavior, due to strong nonlinear interactions, are provided through the system hydrodynamical characteristics. The mechanism leading to the creation of vortex chains results from the combination of the saturation of the radial counterflow instability with the injection of angular momentum in a limited region of space. Let us note that this general behavior was experimentally and theoretically verified to be the same if the number of injected vortices is changed, whether odd or even. We expect that the present scheme will pave the stage to study a series of new vortex collective phenomena that have been observed in cold atom BEC^2^. It is also a first step towards a new class of experiments investigating self-arranged same-sign vortex lattices, unveiling the physics of vortex-vortex interactions in polariton superfluids. In particular, the collective dynamics of the vortex chain (Tkachenko modes)^34^,^35^,^36^, for which the polariton life-time is expected to play a central role in the mode damping, can be of great interest.

## Methods

**Figure 6:**
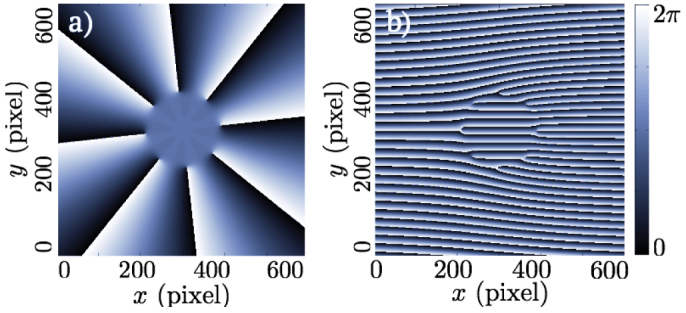
Central flat phase hologram. Hologram corresponding to the method described in the text, without (a) and with (b) the grating. The Laguerre-Gaussian phase is cut at the center and replaced with a flat phase, which creates the Gaussian central beam.

Collimated Laguerre-Gauss beams are a class of modes possessing a simple phase profile with radial invariance. The phase profile of a 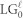 mode is given by the function *φ*_LG_(*r*, *φ*) = *ℓφ* [2*π*], where (*r*, *φ* ∈ [0, 2*π*]) are the polar coordinates in the SLM plane. However, imposing this phase on the SLM produces a superposition of 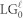 and weak higher modes in the condenser lens focal plane. Since the higher modes are spatially more extended, they can be cut with an iris placed before the condenser lens, which solves the problem. The result is a good quality 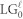 beam. Adding the central Gaussian pump is straightforward as the mode imprinted on the SLM is Gaussian: we simply need to impose a flat phase in the 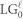 hologram. This has been done by using the phase function *φ*_LG + G_(*r*, *φ*) = *ℓφH*(*r* − *r*_0_) + *π*(1 − *H*(*r* − *r*_0_)), where *H*(*r*) is the Heaviside distribution and *r*_0_ is a constant determining the LG/G intensity ratio. The resulting hologram is presented in Fig. 6 (a).

A small fraction of the light is not modified by the SLM, for which it acts like a mirror. To separate this from the desired mode a grating hologram (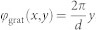, where d controls the separation angle) is added to the LG + G hologram (mathematical sum modulo 2*π*). This vertically deviates the first order reflection forming the pump, allowing us to block the zero-order reflection. The final hologram is presented in Fig. 6 (b).

## Author Contributions

T.B. performed the experiment, analyzed the data and wrote the manuscript. H.T. and D.S. performed the simulations and wrote the manuscript. Q.G. and E.G. participated to the manuscript. A.B. supervised the experiment. G.M. developed the theoretical model. All authors reviewed the manuscript.
